# Challenges and Considerations When Balancing the Risks of Contaminants with the Benefits of Fruits and Vegetables for Infants and Toddlers

**DOI:** 10.3390/nu10111572

**Published:** 2018-10-24

**Authors:** Cheryl Callen, Jatinder Bhatia, Laura Czerkies, William J. Klish, George M. Gray

**Affiliations:** 1 Nestlé Nutrition, Florham Park, NJ 07932, USA; laura.czerkies@us.nestle.com; 2 Department of Pediatrics: Neonatology, Medical College of Georgia, Augusta, GA 30912, USA; jatindeb@augusta.edu; 3 Pediatrics-Gastroenterology, Baylor College of Medicine, Houston, TX 77030, USA; wjklish@hotmail.com; 4 Department of Environmental and Occupational Health, Milkin Institute School of Public Health, George Washington University, Washington, DC 20052, USA; gmgray@gwu.edu

**Keywords:** baby food, contaminants, complementary feeding, risk assessment, nutrient intake

## Abstract

Background: Fruits and vegetables are key to a healthy diet, particularly in children; however, parents may be concerned about contaminants found in fruits and vegetables. Making informed food choices for children requires understanding and balancing the risks of contaminant exposure with the importance of providing a healthy diet. The objective of this work is to identify fruits and vegetables commonly consumed by infants and toddlers; identify potential contaminants in fruits and vegetables; and outline considerations in assessing contaminant risks in food categories with a critical role in a healthy diet. Method: Commonly consumed fruits and vegetables were obtained from the Feeding Infants & Toddlers Study (FITS 2016). The US Food and Drug Administration Total Diet Study was reviewed for contaminant occurrence, and multiple experts were consulted on considerations in assessing risk of certain contaminants. Results: FITS data show eight fruits and nine vegetables account for over 80% of consumption in infants and toddlers. Several contaminants have been detected in fruits and vegetables. Questions to be addressed prior to establishing contaminant guidance were identified. Conclusion: Contaminant guidance for fruits and vegetables consumed by infants and toddlers raises several challenges. Expertise from multiple disciplines is required to find an approach that maximizes public health benefit.

## 1. Introduction

In a 2017 survey of 1002 adult Americans, over 30% were not confident in the safety of the US food supply, and concerns over carcinogens in foods increased from 2016 to 2017 [1]. These concerns may be even greater for parents when selecting and preparing foods for their young children. Questions arise around which foods are best, how to safely prepare them, when to be concerned and what steps to take to ensure the safety of food. Parents need to be well informed to ensure the proper nutrition and safety of foods for their children.

Potentially toxic compounds may be present in raw agricultural crops, including fruits and vegetables, because of natural exposure from soil and water or use of agricultural chemicals. When unintentionally present, these compounds are often referred to as contaminants [2]. Pesticides are also potentially toxic compounds and when present in foods are referred to as pesticide residues. Contaminant levels in crops can be affected by many factors such as environmental conditions and growing practices, including soil type, weather conditions, crop variety and rotation, and harvest and storage practices and conditions [3,4,5,6,7,8]. Preparation, processing, and cooking of fruits and vegetables, whether done at home or commercially, may also result in formation or concentration of contaminants.

Exposure to food contaminants is an important consideration for infants and young children as they may be more susceptible than adults to adverse effects in some cases [9,10,11]. Infants and young children may have a greater intake of specific foods (and their contaminants) per unit body weight [12]. The immaturity of the organ systems in this population may put them at increased risk of negative effects of certain contaminants during sensitive periods of development which could have long-term consequences. Conversely, the infant or young child may also be more resistant to the effects of certain toxic materials during this period depending on the nature, mechanism, and excretory pathway of the material [12,13].

A healthy dietary pattern with higher intakes of fruits and vegetable has been associated with reduced risk of cardiovascular disease, Type II diabetes, certain cancers as well as overweight and obesity [14,15,16]. Based on published data from the 2008 Feeding Infants and Toddlers Study (FITS), fruits and vegetables comprise a significant portion of the infant diet, accounting for 11% of energy for infants 6–8.9 months and 13% of energy for infants 9–11.9 months, second only to the energy contributed by breastmilk and infant formula [17]. Data from FITS 2008 also showed nearly two thirds of children 6–23.9 months of age consumed at least one vegetable per day and about two thirds of children 6–23.9 months consumed fruit, excluding juice, on the day of the survey [18].

There are few specific government standards or guidelines in the United States for contaminant levels in foods for infants and young children. A few notable exceptions include pesticide residue tolerances, maximum lead levels and inorganic arsenic levels in certain fruit juices, and guidance on inorganic arsenic amounts appropriate in infant rice cereals. This may contribute to parents’ alarm when they hear reports of contaminants being found in foods they feed their children. Parents may stop feeding these foods, limit the variety of fruits and vegetables or turn to experiential and anecdotal recommendations such as selecting produce from local farm stands or farmers’ markets or feeling compelled to only buy organic produce for their children with the assumption that these foods would be safer [19]. Although consumption of organically grown crops may have the potential to reduce exposure to pesticide residues compared to conventionally grown crops [20,21,22], the US Department of Agriculture (USDA) Organic program does not define or specify levels for contaminants beyond those defined for conventionally grown crops [23]. Some studies have shown no significant difference for certain contaminants compared to conventionally grown vegetables [24].

Some baby food manufacturers take active steps to control and reduce contaminant levels in fruits and vegetables by investing in agricultural programs and farmer relationships and diligent monitoring of growing, sourcing and processing activities [25]. These steps can contribute to ensuring that crops grown and used for baby foods achieve low levels of contaminants and pesticide residues. For homemade baby foods, parents can help lower contaminant levels and pesticide residues in produce by washing and peeling fruits and vegetables before preparing food for their children. Regardless of these efforts, there is no consensus that these steps are adequate to protect the health and well-being of young children. A resulting question is whether formal contaminant guidance levels or mitigation and control practices should be developed for fruits and vegetables consumed by young children.

The objectives of this publication are: (1) identify the most commonly consumed fruits and vegetables in early childhood; (2) identify some contaminants reported to occur in fruits and vegetables; (3) outline the questions, challenges, and considerations to be addressed in creating contaminant guidance for fruit and vegetable foods for infants and young children; and (4) issue a call to action for key stakeholders. This publication highlights the challenges and considerations in addressing seemingly disparate goals of reducing contaminant exposure from fruits and vegetables consumed by infants and toddlers with the goal of increasing fruit and vegetable consumption for their nutritional and health benefits in a singular comprehensive perspective.

## 2. Materials and Methods

### 2.1. Feeding Infants and Toddlers Study (FITS 2016)

The FITS 2016 was used to assess food consumption among infants 6–11.9 months and toddlers 12–23.9 months in the US. FITS is a cross-sectional, dietary intake study of caregivers of children 0–48 months living in the US [26]. Dietary intake surveys, including a 24-h recall and feeding practices questionnaire, were completed by telephone with 3248 parents and caregivers of children from birth to four years to gain a robust understanding of children’s feeding practices. It is the largest cross-sectional dietary intake survey to date in the US focused on young children, including infants 0–11.9 months (*n* = 1502) and toddlers 12–23.9 months (*n* = 1133).

Methodology for FITS 2016 data collection and analyses have been previously published [26,27]. Briefly, all foods and beverages reported in the 24-h dietary recalls were assigned to food groups. The reported estimated amount of specific foods was calculated on the basis of a single 24-h dietary recall. SAS software (version 9.3; SAS Institute, Cary, NC, USA) and SAScallable SUDAAN^®^ software (release 11; RTI International, Research Triangle Park, NC, USA) were used to incorporate sample weights and produce point estimates and SEs that reflect the US population.

### 2.2. Fruit and Vegetable Consumption

FITS 2016 was used to assess the energy and nutrient contribution of various food categories to the overall diet and evaluate whether a focus on fruits and vegetables consumed by young children may be a method to potentially reduce dietary exposure of certain contaminants. FITS 2016 was also used to identify specific fruits and vegetables consumed, the amount consumed, and whether the fruit or vegetable was a baby food or non-baby food source. Baby food versions are defined as commercially prepared fruits or vegetables (i.e., purees packaged in jars, tubs, or pouches); non-baby food versions included all other forms such as fresh, frozen, or canned fruits and vegetables not specifically marketed for infants or young children. All forms of fruit and vegetables preparation are included such as baked, boiled, or fried. Intakes were assessed to determine the most commonly consumed fruits and vegetables by 6–8.9, 9–11.9, and 12–23.9-month-olds.

The amounts of each food type consumed in a day were based on the average grams reported in the 24-h recall. The mean values reported are a total of each food type, which could have been consumed in one or multiple eating occasions. Fruits and vegetables as a component of a primarily grain, meat/poultry, or dairy-based mixed dish e.g., tomato sauce in lasagna, were not included in the intake assessment.

### 2.3. Contaminant Identification

To identify certain contaminants present in fruits and vegetables, the US Food and Drug Administration Total Diet Study (FDA TDS) was the primary source reviewed to determine the occurrence and levels of elemental contaminants in fruit and vegetables from both baby food and non-baby food sources [28]. A focus on elemental and process contaminants was selected to limit the scope of the analysis. Therefore, potential contaminants from packaging or preparation dishes (e.g., microwavable bowls/cups) were excluded from consideration. Individual pesticide residues and industrial chemicals were also excluded. US FDA recently published results from their 2016 Pesticide Monitoring Program indicating that 99% of domestic products and 90% of imported products were compliant with US standards [29]. Since established pesticide residue limits in the US already consider specific impacts on infants and young children, pesticide residues were not the primary consideration for this publication focused on additional guidance levels. The US FDA also samples foods for presence of mycotoxins, specifically alflatoxins, fumonisins, patulin, ochratoxin A and deoxynivalenol; the results are not reported as part of TDS and are not in scope for this paper. This could be a subject of additional analysis in the future.

The TDS is an on-going program that began in 1961, monitoring for pesticide residues, industrial chemicals, toxic and nutrient elements and radionuclides. This program monitors levels of about 800 contaminants and nutrients in the average US diet. Approximately 280 different kinds of foods and beverages are purchased from around the country from retail outlets as consumers would, prepared, and analyzed for their contaminant and nutrient content four times a year. The list of tested foods, which includes baby foods, is updated about every 10 years to accommodate changing eating patterns over time based on national surveys of food intake conducted by the USDA and the Centers for Disease Control (CDC). A central US FDA laboratory prepares the food samples for analysis, and samples from the three cities of each region are combined. Analyses are done using standardized analytical methods that are updated as needed [28].

### 2.4. Expert Consultations

To identify the key challenges, questions, and considerations in developing contaminant guidance for fruits and vegetables for infants and young children, we consulted experts from a broad range of disciplines including pediatricians, nutritionists, toxicologists, agricultural and agronomists, and food scientists. The pediatricians consulted had expertise in neonatology, gastroenterology, and nutrition. The toxicologists consulted have expertise in risk assessment, environmental contaminants, and exposure assessments as well as regulatory expertise. Agricultural experts, food scientists and agronomists who were consulted have expertise in procurement, supply chain management, crop rotation and pest management, general agricultural practices in produce production and food manufacturing.

Topics included the nutritional importance of fruits and vegetables in the diet of young children, sources of contaminant exposure beyond fruits and vegetables, risk mitigation practices and communications to consumers. The experts also contributed to the outline for a systematic approach to addressing the question of whether contaminant guidance should be established for fruits and vegetables consumed by infants and young children.

## 3. Results

### 3.1. Food Category Selection

Based on FITS 2016, breastmilk and infant formula are the major source of energy for infants (0–12 months). Breastmilk and infant formula intake are accounted for in the Milk/Milk products grouping. Fruits and vegetables, inclusive of both baby food and non-baby food sources, are the second highest contributor to energy intake at about 14% of calories per capita for infants 6–11.9 months. Grain products comprise the next highest food category in terms of energy contribution for infants. For toddlers (12–23.9 months), fruit, 100% fruit juice and vegetables continue to contribute significantly to the diet, comprising 12.2% to 18.6% of energy intake per capita and competing with grains and grain products as the second highest contributor to energy intake after cow’s milk (Table 1).

For infants 6–11.9 months of age, FITS 2016 shows baby food fruits and vegetables are among the top five food sources for dietary fiber, potassium, and vitamin E and baby food vegetables are among the top five food sources for iron. For children 12 to 23.9 months, among the top five food sources for potassium are fruit (14% of intake) and vegetables (5% of intake); among the top five food sources for vitamin E are fruits (7% of intake) and vegetables (6% of intake); and fruits and vegetables are the number 1 and number 2 food source for dietary fiber contributing 23% and 10% of intake, respectively.

### 3.2. Primary Fruit and Vegetable Varieties Consumed

Managing contaminant levels in the fruits and vegetables consumed in the largest amounts may be an approach to reducing contaminant exposure in infants and young children. The data indicate that commercially available baby food versions of most fruits and vegetables are the most common forms eaten up until 1 year of age (Table 2 and Table 3). While a variety of fruits and vegetables are reported as consumed by infants and young children, the predominant vegetables consumed by children under 12 months of age are sweet potatoes and white potatoes, carrots, green beans, squash, peas, and corn. These vegetables and mixtures thereof account for over 80% of total vegetable intake on a per capita basis. The predominant fruits consumed under 12 months of age are apples, bananas, pears, peaches, oranges, avocado and mixtures of these. White potatoes contribute more significantly to vegetable intake between 12 and 23.9 months, accounting for over 30% of total vegetable intake on a per capita basis compared to 12% for infants 9–11.9 months of age.

### 3.3. Contaminant Occurrence

Reported levels from TDS [28] of elemental and process contaminants included in the scope of the analysis are provided in Table 4 for select fruits and vegetables, both as tested in baby food items and non-baby food items. Mean values in mg/kg food (ppm), the standard deviation, and minimum and maximum levels are summarized.

For ease of understanding, the values from Table 4 [28,30] are described in ppb (mcg/kg) for each selected contaminant in the following descriptive section:

Lead: In the US FDA TDS, lead was detected in several fruit and vegetable products including baby food fruit and vegetables [28]. Among the fruit and vegetables analyzed, baby food sweet potatoes had the highest mean level reported at 13 ppb (high value 34 ppb). Lead levels were comparable for non-baby food sweet potatoes (mean level 12 ppb; maximum level reported 23 ppb). Avocado, squash, green beans, and carrots were also reported to have detectable levels of lead. Fruits and vegetables with the lowest levels of lead reported were bananas, peaches, pears, and peas.

Cadmium: Leafy vegetables such as lettuce and spinach, potatoes and grains, peanuts, soybeans, and sunflower seeds have been reported to contain approximately 50–120 ppb cadmium [31]. Based on the US FDA TDS, the highest value reported for cadmium was in spinach (1100 ppb) with a mean value of 183 ppb. Carrots and potatoes were next highest with mean levels of 19 ppb and 31 ppb, respectively and maximum levels of 62 ppb and 65 ppb. Cadmium was detected in several other fruits and vegetables including cauliflower, broccoli, strawberries, and squash [28].

Arsenic: Many fruits and vegetables were reported to have non-detectable levels of arsenic in the US FDA TDS, including apples, bananas, peas, green beans, carrots, and sweet potatoes [28]. The fruits and vegetables with higher reported levels included peaches, potatoes, pears, squash, grapes, strawberries, tomatoes, broccoli, corn, and spinach with reported values ranging between 8 and 18 ppb. Avocado had the highest reported value in this category at 44 ppb.

Mercury: The US FDA TDS reports few mercury levels for fruit and vegetable products and those reported were either non-detectable or quite low (<1 ppb) [28]. Levels of total mercury in vegetables are generally low (<10 ppb) based on data reported by the European Food Safety Authority (EFSA) on samples collected between 2002 and 2011 across 20 European countries. The exception was mushrooms sourced from France and Germany [32].

Perchlorate: The US FDA TDS reported perchlorate levels in baby food fruits and vegetables ranging from 0–16 ppb in baby food fruits to 1–74 ppb for baby food vegetables. For non-baby food fruits and vegetables the highest levels reported were for: grapes (mean 25.83 ppb; high value 90.6 ppb), spinach (mean value 16.79 ppb; high value 88 ppb), raw tomato (mean value 57.8 ppb; high value 88 ppb), broccoli (mean value 10.7 ppb; high value 102 ppb), and summer squash (mean value 32.2 ppb; high value 197 ppb). Collard greens had a level reported as high as 1090 ppb [33]. A recent study indicates children have higher levels of perchlorate intake than adults [34].

Furan: Furan may form in foods during traditional heating methods, such as cooking and canning. Through 2004, the US FDA conducted exploratory testing of furan levels in food, including baby food. Levels of furan in baby food fruits and vegetables ranged from about 3 ppb in applesauce to 108 ppb in sweet potatoes [35].

Acrylamide: The US Food and Drug Administration reported acrylamide levels in foods as part of the TDS from 2003–2006. The majority of the 56 baby foods sampled in 2003 were non-detectable for acrylamide (38 samples or 68%). In the 2004–2006 TDS, sampling focused on baby foods that had detectable results in 2003. The highest levels reported for acrylamide in baby food fruits and vegetables included sweet potatoes, carrots, and squash [28]. Baby food varieties of prunes and prune juice were not tested in the US FDA Total Diet Study. However, bottled prune juice was tested in 2003, 2004, 2005, and 2006 with results ranging between 109 and 355 ppb.

### 3.4. Considerations, Questions and Challenges Identified

The nutrients and energy from fruits and vegetable have clear health benefits while contaminants may pose health risks therefore, managing the issue of contaminants of potential concern in foods for infants and young children is a classic “risk vs. risk” challenge [36,37,38]. The experts consulted raised several difficult questions, considerations, and challenges for the development of contaminant guidance for fruits and vegetables for infants and young children.

- Which contaminants are of most concern to infants and young children?
  ○ Has exposure to the contaminant in early childhood been reported to have negative effects on growth and development beyond the health effects reported in adults?
  ○ Is the contaminant present at detectable and quantifiable levels of concentration in the fruits and vegetables commonly consumed by infants and young children?
- What is the risk posed by these contaminants?
  ○ Is there knowledge of a dose-response relationship between exposure and harm?
  ○ How do exposure levels compare to standard toxicologic benchmarks?
- What are the health benefits of fruits and vegetables?
  ○ Is there a dose-response relationship between intake and benefit?
  ○ What are current and desired levels of intake?
- How to balance/characterize the risk tradeoff?
  ○ How can disparate outcomes be compared (e.g., obesity and heart disease vs. possibility of subtle neurologic effects)?
  ○ Are the magnitude of risks and benefits understood?
- How can contaminant risks be managed or mitigated?
  ○ Can contaminant risks be managed or mitigated through agricultural and/or processing practices?
  ○ Should feeding recommendations be altered to manage or mitigate risks (e.g., limiting amounts or types of certain fruit or vegetable varieties)?
- How best to communicate with consumers?
  ○ What is the best way to communicate risk tradeoffs to consumers?
  ○ What is the goal in informing the consumer—what changes in choices or behaviors is desired?

## 4. Discussion

Adequate consumption of fruits and vegetables is the core of a healthy diet for children and adults and is an area of emphasis in the Dietary Guidelines for Americans. The American Academy of Pediatrics (AAP) recommends offering a fruit or vegetable at every meal and snack starting at 6 months of age [39]. Exposure to a variety of fruits and vegetables in early childhood may affect food preferences and these preferences may continue into older childhood [40].

Fruits and vegetables in general are known for their high nutrient density and low energy density, which makes them particularly beneficial in early childhood nutrition. Diets with higher amounts of fruits and vegetables are also recognized for long-term health benefits. Consumption of fruits and vegetables at the recommended amounts are associated with lower risk of coronary heart disease, lower incidence of overweight and obesity and an association with lower risk of morbidities such as Type II diabetes and certain cancers [41,42,43]. Additionally, factors such as physical activity, lifestyle, socio-economic factors, genetics, and epigenetics can significantly modify outcomes associated with fruit and vegetable consumption.

Understanding the significant contribution fruits and vegetables make to the infant and toddler diet, it is reasonable to consider a focus on these foods as a potential source of contaminant exposure in the diets of young children. Consideration of the importance of fruit and vegetable intake to long-term health and to the role of fruit and vegetable intake early in life regarding shaping food preferences is essential especially if suggested guidance levels could effectively exclude certain fruit and/or vegetable varieties from the diet.

Contaminant intake risk is commonly evaluated based on impact to health and disease risk (e.g., cancer risk) over a lifetime of exposure. When considering contaminant exposure in infants and young children specifically, the impact to health and disease risk should also focus on acute or short term exposure during this period of life. Acute risks are not typically expected from exposure to low levels of contaminants in foods. As a result, determining which contaminants are most important will require a disciplined approach including alignment on the following key questions: (1) what process and criteria should be used for selecting contaminants of concern, including the level of evidence required to suggest a negative impact on infants and young children beyond what is expected for adults and older children; (2) in the absence of a sufficient level of evidence, what research gaps need to be filled; (3) while evidence is being generated, what guidance, if any, is warranted; (4) based on mechanism of the negative effect, should contaminants be considered individually or collectively.

The mere presence of a contaminant in fruits and vegetables does not suggest the food is unsafe. To determine the risk requires knowledge of the dose-response relationship and level of exposure. If an acceptable daily intake (ADI) or tolerable daily intake (TDI) exists, a “safe” level can be determined that potentially minimizes risks from a specific food based on estimated intake. However, this may not be an effective approach when no “safe” dose is recognized, and absence of the contaminant cannot be assured or when the levels naturally present in the food do not meet the estimated “safe” level.

The As Low as Reasonably Achievable (ALARA) Principle is often suggested as an approach when no safe level can be established, but this approach can be subject to interpretation. A key question when using the ALARA principle is whether an interim benchmark level should be established for purposes of monitoring and measuring improvement. Levels of the contaminant reported to be found in specific fruit and vegetable varieties [28] can serve as a reference point for identifying specific varieties of fruits and vegetables that may require a priority focus.

Finally, controlling the contaminant may need to be done across both baby food and non-baby food forms of fruit and vegetables to adequately reduce exposure and risk for potential negative effects given that baby food fruit and vegetables are not the only form of fruits and vegetables consumed, even under 1 year of age.

**Figure 1:**
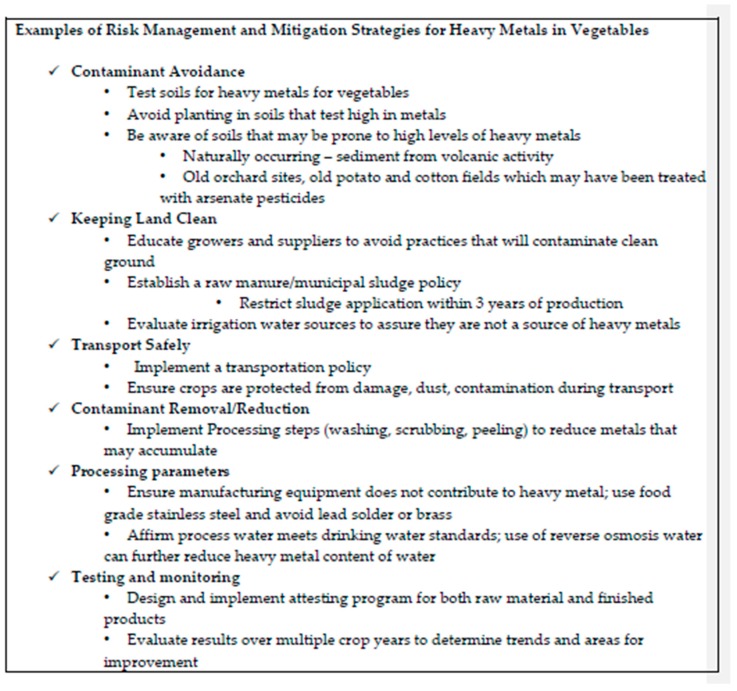
Practices Used to Control Heavy Metals in Baby Food Vegetables.

Agricultural experts, farmers, growers, and food manufacturers, including baby food manufacturers, would need to partner to identify the potential sources of contaminants in various fruit and vegetables (e.g., soil, water, processing, cleaning agents, agricultural chemicals) and recommend tools and techniques for use by growers, processors and food manufacturers to control and reduce the source of the contamination. An outcome might be the development of a Code of Practice similar to the Codex Alimentarius Code of Practice for the Prevention and Reduction of Lead Contamination of Foods [43] or guidance similar to that developed by the US FDA for reducing acrylamide formation in foods [44]. Other examples of practices used to control heavy metals in baby food vegetables are shown in Figure 1. Programs such as the one described may take years to implement and consistent results may not be assured for several growing seasons. Variability in uptake of contaminants can occur based on environmental conditions making testing and monitoring over time useful in measuring whether actions are effective.

Despite the best practices and controls, fruits and vegetables may vary in the level of certain contaminants based on factors that may not be avoidable, such as weather conditions. Contaminant guidance levels if created, would need to be applied in ways that discourage unnecessary waste of crops, produce or ingredients that are acceptable and safe to consume. Determining how guidance levels could be applied without creating unnecessary waste should be a key consideration.

After the above questions and considerations are addressed there remain challenges in determining how best to communicate risks and benefits to parents, caregivers, and health care professionals. This is especially important in a food category understood to be a pivotal component of a healthy diet that reduces long-term risk for disease and which may contribute nutrients or other nutritive substances protective of health [37,45]. Guidance should be holistically encompassing for all forms of fruits and vegetables fed to baby to help parents make informed feeding choices. It should be applicable to commercial baby foods as well as fruits and vegetables made at home or purchased in other forms (e.g., fresh, frozen, and canned). Isolating any of these feeding styles may result in food avoidance or discourage the variety of fruits and vegetables necessary for a healthy diet.

Moreover, it is challenging to communicate risk without creating unnecessary concern or non-preferred dietary behaviors (e.g., avoidance of certain fruit and vegetables). Communication experts should be consulted on the appropriate ways to communicate the management of contaminant risks to parents and caregivers while reinforcing the benefits of feeding these foods to their children. Alignment among stakeholders in developing and reinforcing a consistent message to parents about the nutrition and safety of the foods they feed their children should be of paramount importance.

Clearly, the consideration of contaminant guidance for fruits and vegetables for infants and young children poses a unique challenge but also opportunity for debate and consensus building among stakeholders from multiple disciplines including toxicology, nutrition, risk assessment, agriculture, food processing, regulatory, and risk communication. There are several ways this discussion could be advanced including through public-private partnerships; that involve academia, government, industry, and consumer advocacy groups.

## 5. Conclusions

Advancing the conversation to help characterize and manage contaminant risks for infants and young children in the major food categories, fruits, and vegetables, would provide transparency and ensure these foods are safe and protective of infant health. Focusing on fruits and vegetables as a first step is justified based on their energy and nutrient contribution to the diet of young children and the potential to also be a dietary source of certain heavy metals and other contaminants. The risks of potential consequences such as increased risk of obesity and heart disease associated with diets lower in fruits and vegetables will need to be weighed against potential risks associated with contaminant exposure.

Overall, there are challenging questions to be answered regarding whether contaminant guidance is needed or beneficial, and we hope this sparks conversation and a call to action among multiple stakeholders to develop an aligned approach for identification and management of risks, including risks of inadequate intake and loss of variety, for fruits and vegetables in the diets of infants and young children. These conversations can enhance the overall safety of foods that comprise a significant component of the diet, reassure parents, and ultimately help to ensure adequate consumption and variety of fruits and vegetables in the diet of infants and young children.

## Figures and Tables

**Table 1:** Food sources of energy (calories per capita per day) from major food groups among children 6–23.9 months: FITS 2016.

Age Groups	6–8.9 Months	9–11.9 Months	12–14.9 Months	15–17.9 Months	18–20.9 Months	21–23.9 Months
	*n* = 468	*n* = 434	*n* = 412	*n* = 308	*n* = 251	*n* = 162
	Kcal/day (% of total daily energy)					
Total daily energy	769	930	1045	1156	1216	1167
Milk/milk products	552 (71.8)	520 (55.9)	361 (34.5)	345 (29.8)	302 (24.8)	253 (21.7)
Grains/grain products	60 (7.8)	112 (12)	177 (16.9)	179 (15.5)	205 (16.9)	219 (18.8)
Mixed dishes	16 (2)	55 (5.9)	110 (10.5)	140 (12.1)	161 (13.2)	136 (11.7)
Meat and other Protein sources	19 (2.5)	58 (6.2)	130 (12.4)	165 (14.3)	182 (14.9)	168 (14.4)
Vegetables	9 (1.2)	21 (2.3)	19 (1.8)	24 (2.1)	22 (1.8)	20 (1.7)
White potatoes	7 (0.9)	10 (1.1)	25 (2.4)	27 (2.3)	29 (2.4)	33 (2.8)
Baby food vegetable	24 (3.1)	21 (2.3)	5 (0.4)	4 (0.3)	2 (0.2)	1 (0)
Fruit	13 (1.7)	35 (3.8)	64 (6.1)	70 (6.1)	83 (6.8)	94 (8.1)
Baby food fruit	38 (1)	37 (3.9)	13 (1.2)	7 (0.6)	5 (0.4)	3 (0.3)
100% juice	12 (1.6)	25 (2.7)	35 (3.3)	48 (4.2)	58 (4.8)	66 (5.7)
Sweets and desserts	12 (1.6)	34 (3.7)	87 (8.3)	118 (10.2)	134 (11)	133 (11.4)
Savory snacks	3 (0.3)	6 (0.6)	11 (1.1)	18 (1.6)	15 (1.2)	27 (2.3)
Fats	2 (0.3)	3 (0.3)	4 (0.4)	8 (0.7)	10 (0.8)	8 (0.7)

**Table 2:** Top Fruits Consumed by Age Range in Mean grams Per Capita: FITS 2016 *.

	6–8.9 Months (Mean 7.6 ± 0.9 Months) Mean g/Capita/Day *n* = 468		9–11.9 Months (Mean 10.5 ± 0.9 Months) Mean g/Capita/Day *n* = 434		12–23.9 Months (Mean 16.9 ± 3.3 Months) Mean g/Capita/Day *n* = 1133	
Total fruit mean g/capita/day	77 g		110 g		127 g	
FRUITS	Baby Food	Non-Baby food	Baby Food	Non-Baby food	Baby Food	Non-Baby food
Apples/apple mixtures	24	5	27	10	5	34
Banana/banana mixtures	16	6	13	18	2	30
Pears/pear mixtures	10	3	11	3	3	3
Peaches				3		5
Avocado				3		2
Strawberry						5
Oranges				3		11
Grapes						7
% of total fruit	83%		83%		84%	

* Grayed out boxes indicate a fruit was not counted toward the top 80% of fruit consumed in that age group. Mixtures have more than then one ingredient and are categorized by the most prominent ingredient.

**Table 3:** Top Vegetables Consumed by Age Range in Mean grams Per Capita: FITS 2016 *.

	6–8.9 Months (Mean 7.6 ± 0.9 Months) Mean g/Capita/Day *n* = 468		9–11.9 Months (Mean 10.5 ± 0.9 Months) Mean g/Capita/Day *n* = 434		12–23.9 Months (Mean 16.9 ± 3.3 Months) Mean g/Capita/Day *n* = 1133	
Total vegetables mean g/capita/day	76 g		73 g		64 g	
Vegetables	Baby Food	Non-Baby food	Baby Food	Non-Baby food	Baby Food	Non-Baby food
Sweet potatoes/mixtures	14	6	12	4	0.8	2
Carrots/mixtures	10	2	8	3	1	4
Squash/mixtures +	8	1	8	0.7	1	0.5
Peas/Mixtures	4	1	5	1	0.1	2
Green Beans/mixtures	10	1	5	3	0.5	6
White potatoes	--	6	--	9	--	20
Corn/mixtures	2	0.1	1	0.6	0.3	4
Tomatoes					--	5
Broccoli					0.2	5
% of total vegetable	86%		83%		82%	

* Grayed out boxes indicate a vegetable was not counted toward the top 80% of vegetables consumed in that age group. -- indicates these vegetables are not a predominant ingredient in baby foods. Mixtures have more than then one ingredient and are categorized by the most prominent ingredient. + Squash (all types) includes summer squash, zucchini, butternut squash, acorn squash.

**Table 4:** Summary of US FDA Total Diet Study Results for Arsenic, Cadmium, Lead, Mercury and Perchlorate in Select Fruits and Vegetables [28,30].

	Mean Level ± Standard Deviation (min, max) (mg/kg Food) *				
Food	Arsenic	Cadmium	Lead	Mercury **	Perchlorate ^+^
BF carrots	0 ± 0 (0,0)	0.017 ± 0.012 (0,0.059)	0.004 ± 0.006 (0,0.018)		0.010 ± 0.018 (0,0.074)
Carrots (fresh, peeled, boiled)	0 ± 0 (0,0)	0.019 ± 0.015 (0.002,0.062)	0.002 ± 0.004 (0,0.019)		0.004 ± 0.003 (0,0.010)
BF Green Beans	0 ± 0 (0,0)	0.001 ± 0.001 (0,0.003)	0.001 ± 0.005 (0,0.021)		0.004 ± 0.002 (0,0.009)
Green Beans (fresh, frozen/boiled)	0 ± 0 (0,0)	0.001 ± 0.002 (0.006)	0.001 ± 0.003 (0,0.018)		0.009 ± 0.014 (0,0.059)
BF sweet potatoes	0 ± 0 (0,0)	0.004 ± 0.002 (0,0.005)	0.013 ± 0.008 (0,0.034)		0.001 ± 0.001 (0,0.003)
Sweet potatoes (canned)	0 ± 0 (0,0)	0.004 ± 0.002 (0,0.007)	0.012 ± 0.005 (0,0.023)		0.0004 ± 0.001 (0,0.003)
Potato, boiled (without peel)	0.0003 ± 0.002 (0,0.010)	0.021 ± 0.008 (0.01,0.039)	0 ± 0 (0,0)		0.001 ± 0.001 (0,0.003)
Potato, baked (with peel)	0.001 ± 0.003 (0,0.012)	0.031 ± 0.013 (0,0.065)	0.0004 ± 0.002 (0,0.012)		0.0004 ± 0.001 (0,0.002)
BF applesauce	0 ± 0 (0,0)	0.001 ± 0.002 (0,0.007)	0.001 ± 0.002 (0,0.009)		0 ± 0 (0,0)
Applesauce	0 ± 0 (0,0)	0.0004 ± 0.02 (0,0.008)	0.001 ± 0.003 (0,0.011)		0 ± 0 (0,0)
Apples (raw, with peel)	0 ± 0 (0,0)	0.001 ± 0.004 (0,0.022)	0 ± 0 (0,0)		0.0001 ± 0.0002 (0,0.001)
BF Peaches	0.001 ± 0.002 (0,0.010)	0.003 ± 0.001 (0,0.006)	0.0003 ± 0.001 (0,0.008)		0.005 ± 0.004 (0.001,0.016)
Peaches (raw, frozen)	0.0003 ± 0.002 (0,0.009)	0.002 ± 0.002 (0,0.006)	0.0002 ± 0.001 (0,0.007)		0.001 ± 0.002 (0,0.009)
BF peas	0 ± 0 (0,0)	0.002 ± 0.002 (0,0.005)	0.0002 ± 0.001 (0,0.007)		0.0002 ± 0.0004 (0,0.001)
Peas (green, frozen, boiled)	0 ± 0 (0,0)	0.002 ± 0.002 (0,0.008)	0 ± 0 (0,0)		0.0003 ± 0.0007 (0,0.003)
BF Pears	0 ± 0 (0,0)	0.002 ± 0.002 (0,0.004)	0.0003 ± 0.001 (0,0.008)	0 ± 0 (0,0)	0.0001 ± 0.0005 (0,0.002)
Pears (raw, with peel)	0.0003 ± 0.002 (0,0.010)	0.001 ± 0.002 (0,0.006)	0.001 ± 0.002 (0,0.009)		0.0008 ± 0.003 (0,0.011)
BF Bananas	0 ± 0 (0,0)	0.001 ± 0.002 (0,0.006)	0 ± 0 (0,0)	0 ± 0 (0,0)	0.0001 ± 0.0002 (0,0.001)
Bananas	0 ± 0 (0,0)	0.001 ± 0.001 (0,0.003)	0 ± 0 (0,0)		0.0005 ± 0.0009 (0,0.002)
BF squash	0.0004 ± 0.002 (0,0.013)	0.002 ± 0.002 (0,0.010)	0.001 ± 0.005 (0,0.022)		0.002 ± 0.002 (0,0.005)
Squash, winter (fresh, frozen/boiled)	0 ± 0 (0,0)	0.005 ± 0.006 (0,0.03)	0.001 ± 0.003 (0,0.012)		0.006 ± 0.008 (0,0.032)
Squash, summer (fresh, frozen/boiled)	0 ± 0 (0,0)	0.002 ± 0.002 (0,0.011)	0.001 ± 0.003 (0,0.018)		0.0322 ± 0.055 (0,0.197)
Avocado (raw)	0.001 ± 0.008 (0,0.044)	0.012 ± 0.012 (0,0.054)	0.001 ± 0.005 (0,0.030)	0.0001 ± 0.0003 (0,0.001)	0.004 ± 0.004(0,0.018)
Grapes (red/green, raw)	0.002 ± 0.005 (0,0.018)	0.001 ± 0.001 (0,0.004)	0.002 ± 0.004 (0,0.019)		0.026 ± 0.030 (0,0.091)
Oranges	0 ± 0 (0,0)	0.0004 ± 0.001 (0,0.006)	0.001 ± 0.004 (0,0.021)		0.004 ± 0.004 (0,0.019)
Pineapple (canned in juice)	0 ± 0 (0,0)	0.003 ± 0.002 (0,0.011)	0.007 ± 0.009 (0,0.046)		0.002 ± 0.002 (0,0.005)
Strawberries (raw/frozen)	0.0003 ± 0.002 (0,0.009)	0.015 ± 0.010 (0.003,0.044)	0.001 ± 0.004 (0,0.015)		0.002 ± 0.005 (0,0.020)
Broccoli (fresh, frozen/boiled)	0.0003 ± 0.002 (0,0.010)	0.009 ± 0.005 (0.004,0.025)	0 ± 0 (0,0)		0.011 ± 0.024 (0,0.102)
Cauliflower (fresh, frozen/boiled)	0 ± 0 (0,0)	0.009 ± 0.005 (0.002,0.030)	0.0005 ± 0.003 (0,0.015)	0 ± 0 (0,0)	0.004 ± 0.008 (0,0.036)
Corn (fresh, frozen/boiled)	0.0004 ± 0.002 (0,0.013)	0.002 ± 0.002 (0,0.008)	0 ± 0 (0,0)		0 ± 0 (0,0)
Spinach (fresh, frozen/boiled)	0.0003 ± 0.001 (0,0.008)	0.183 ± 0.226 (0.038,1.1)	0.004 ± 0.005 (0,0.018)	0.0003 ± 0.0005 (0,0.001)	0.017 ± 0.019 (0,0.088)
Tomato (raw)	0.0003 ± 0.002 (0,0.010)	0.008 ± 0.007 (0,0.029)	0 ± 0 (0,0)	0 ± 0 (0,0)	0.058 ± 0.056 (0,0.088)

* The data reported here pertain to TDS market baskets 2006-1 through 2013-4. ** Mercury data includes market baskets 2006-1 through 2008-1 and 2011-4 through 2013-4. Results from 2008-2 through 2009-4 were omitted due to methodology issues. Samples collected from 2010-1 through 2011-3 were not analyzed for mercury. Gray boxes indicate no data were reported for this food. ^+^ Perchlorate data includes market baskets from the 2008–2012 Total Diet Study results. For a particular basket, if the perchlorate level was not detected, a value of 0 parts per billion (ppb) of perchlorate was used in calculating the mean and standard deviation. Note the unit for perchlorate in TDS was reported in ppb unlike the other contaminants in this table and converted to ppm (mg/kg food). Mean and SD were calculated from the raw data for perchlorate for TDS [30].
